# Production, purification and characterization of a novel thermotolerant endoglucanase (CMCase) from *Bacillus* strain isolated from cow dung

**DOI:** 10.1186/2193-1801-2-10

**Published:** 2013-01-12

**Authors:** Sangrila Sadhu, Pradipta Saha, Sukanta K Sen, Shanmugam Mayilraj, Tushar Kanti Maiti

**Affiliations:** 1 Microbiology Laboratory, Department of Botany, Burdwan University, Burdwan, 713104 WB India; 2 Department of Microbiology, Burdwan University, Burdwan, 713104 WB India; 3 Department of Botany, Siksha Bhavana, Visva- Bharati, Santiniketan, 731235 WB India; 4 Microbial type culture collection (MTCC), Institute of Microbial Technology (IMTECH), Sector 39-A, Chandigarh, 160036 India

**Keywords:** Endoglucanase (CMCase), *Bacillus* sp, Thermostable

## Abstract

In an attempt to screen out cellulase producing bacteria from herbivorous animal fecal matter it was possible to isolate a potent bacterium from cow dung. The bacterium was identified as *Bacillus* sp. using 16S rDNA based molecular phylogenetic approach. The effect of different agricultural wastes, paper wastes and carboxymethyl cellulose on endoglucanase production was tested and was found to produce maximally at 8% carboxymethyl cellulose. The endoglucanase was precipitated by ammonium sulfate saturation and purified by DEAE- Sepharose column. The purification was achieved 8.5 fold from the crude extract with a yield of 68.1%. The molecular weight of the protein was determined to be 97 kDa by SDS-PAGE. The enzymatic activity was moderately reduced by detergents (SDS, Tween-80), metal ions (MnCl_2_, ZnCl_2_) and EDTA. The endoglucanase was stable between pH 5.0 – 9.0 and temperature between 20−70°C with optimal activity at pH 7.0 and temperature 50°C. The apparent Km value of the enzyme for the substrate carboxymethyl cellulose was recorded to be 0.25 mg/ml. The endoglucanase was stable in the presence of commercial detergents such as Ariel, Surf Excel and Tide, indicated might be of potential applications in detergent industry. The enzyme from this strain could also be applied in bioconversion of lignocellulosic biomass into fermentable sugars.

## Background

A promising approach relies on the production of bioethanol from the abundant and renewable lignocellulosic biomass (Hahn-Hägerdal et al. 2006). Cellulose, the most common natural renewable biopolymer, is commonly degraded by the hydrolytic action of a multicomponent enzyme system - the cellulase and represents the key step for biomass conversion. The enzymatic hydrolysis requires synergistic action of cellobiohydrolase or exoglucanase (E.C.3.2.1.91), endoglucanase or carboxymethylcellulase (E.C. 3.2.1.4) and cellobiase or β-glucosidase (E.C.3.2.1.21).

Applications of this enzyme such as the production of animal feed, formulation of detergents, juice clarification, paper industry and wine production. Cellulases contribute to 8% of the worldwide industrial enzyme demands and the demand is expected to increase by 100% within 2014 (Costa et al. 2008). For these processes thermophilic and or alkalophillic or acidophilic microorganisms as sources of thermostable and wide range of pH stable enzymes are needed, because of their higher stability and activity over a wider range of temperatures and pH (Bakare et al. 2005; Viikari et al. 2007).

However, bacteria may also serve as a novel source cellulases due to their higher growth rate, more complex glycoside hydrolases providing synergy with higher potency because of organismal diversity of extreme niches. The latter feature provides them capabilities to produce cellulases that can withstand extreme conditions (Miranda et al. 2009) like thermostability, acid and alkalistability, which makes them successful candidate for industrial applications.

Many workers have purified and characterized cellulases isolated from different bacteria viz. *Thermomonospora* sp. (George et al. 2001), *Cellulomonas* sp. YJ5 (Yin et al. 2010), *Melanocarpus* sp. MTCC 3922 (Kaur et al. 2007), *Pseudomonas fluorescens* (Bakare et al. 2005), *Pyrococcus horikoshi* (Kang et al. 2007), *Bacillus* sp (Acharya and Chaudhury 2011; Ashabil et al. 2011, Bajaj et al. 2009, Bischoff et al. 2006, Kim et al. 2005; Singh et al. 2004). The present manuscript deals with the production, purification and characterization of a thermostable endoglucanase from *Bacillus* sp. C1 (MTCC10046), isolated from cow dung. The property of the purified enzyme was studied to understand its potential for biotechnological application.

## Results and discussion

### Isolation and identification of cellulolytic bacteria

**Figure 1:**
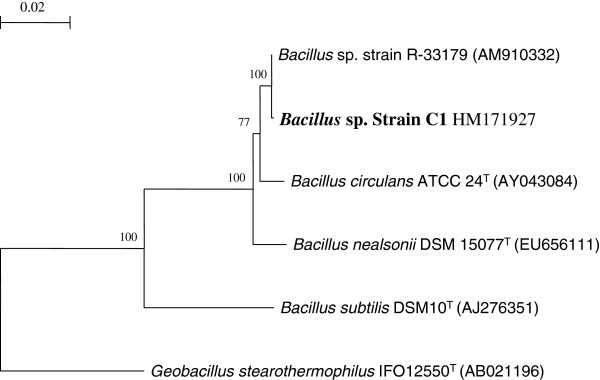
**Phylogenetic tree based on neighbour-Joining method, showing the relationship between strain C1 with other related taxa.** Bootstrap values of 100 replications are shown at the branch. The tree was generated using TREECON (Van de Peer & Wachter, 1997) and Jukes & Cantor (1969) correction. *Sequence from Geobacillus stearothermophilus* strain DSM10^T^ was taken as out group Bar. 0.02 base substitutions per site.

Out of 20 bacterial isolates from cow dung the isolate C1 was selected as potent cellulose hydrolyser using Omeliansky’s agar medium forming clear zone around growth and by cellulase assay with cell free culture filtrate. Cow dung was selected as a source for obtaining desirable cellulase producing organisms, because it is a rich source of diverse group of cellulolytic microorganisms owing to diet of the ruminants which consists of high amounts of cellulosic matter. The isolate was characterized through cultural, morphological, physiological and biochemical studies (data not shown except carbon sources utilization pattern by BIOLOG method in Table 1). The strain was deposited in Microbial Type Culture Collection & Gene Bank (MTCC), Chandigarh, India and its accession number is MTCC 10046. The nucleotide accession number of 16S rDNA is HM171927 obtained from NCBI. Among well established species of the genus *Bacillus*, the strain C1 showed closest sequence identity (99.04%) with *Bacillus circulans* ATCC 24^T^ followed by *Bacillus nealsonii* DSM 15077^T^ (Figure 1). Sequence similarity with type strains of other *Bacillus* Spp. was less than 98%. In the absence of overall genome relatedness, chemotaxonomic data, the strain C1 is identified as *Bacillus* sp.

**Table 1:** **Oxidation of substrates using Biolog GP2 microplates**

Oxidation of substrate using Biolog GP2 microplates	C1	Oxidation of substrate using Biolog GP2 microplates	C1	Oxidation of substrate using Biolog GP2 microplates	C1
α-cyclodextrin	+	maltotriose	+	acetic acid	-
β cyclodextrin	+	D-mannitol	+	α- hydroxybutyric acid	-
Dextrin	+	D-mannose	+	β- hydroxybutyric acid	-
glycogen	+	D-melezitose	-	γ-hydroxybutyric acid	-
Inulin	-	D-Melibiose	-	p-hydroxyphenylacetic acid	-
mannan	-	β-methyl-D-galactoside	-	α-ketoglutaric acid	-
tween-40	-	α -methyl-D-galactoside	-	α -ketovaleric acid	-
tween-80	-	3-methyl-D- glucose	-	Lactamide	-
N-acetyl-D-Glucosamine	+	α -methyl-D-glucoside	-	D-lactic acid Methyl Ester	-
N-Acetyl β-D-mannosamine	-	β -methyl-D-glucoside	+	L-lactic acid	-
amygdain	-	α -methyl-D-mannoside	-	D-Malic Acid	-
L-arabinose	-	palatinose	-	L-Malic Acid	+
D-arabitol	-	D-psicose	-	Pyruvic acid methyl ester	-
Arbutin	+	D-raffinose	-	Succinic Acid mono methyl ester	-
D-cellobiose	+	L-rhamnose	-	Propionic acid	-
D-cellobiose	+	D-ribose	-	pyruvic acid	-
D-fructose	+	salicin	+	Succinamic Acid	-
L-fucose	-	sedoheptulosan	-	Succinic Acid	-
D-galacturonic acid	-	D-sorbitol	+	N-Acetyle L Glutamic Acid	-
D-galactose	-	stachyose	-	L-Alaninamide	-
gentiobiose	-	sucrose	+	D-Alanine	-
D-gluconic acid	-	D-tagatose	-	L-Alanine	-
α –D-glucose	+	D-trehalose	+	L-Alanyl-Glycine	-
m-inositol	-	Turanose	-	L-Asparagine	-
α -D- lactose	-	Xylitol	-	L-Glutamic Acid	-
lactulose	+	D-xylose	+	Glycyl-L-Glutamic Acid	-
maltose	+	L-Pyroglutamic Acid	-	uridine	-
L-Serine	-	adenosine	-	Adenosine 5’-monophosphate	-
Putrescine	-	2-deoxy adenosine	-	Thymidine-5’-Monophosphate	-
2-3 butenediol	-	inosine	-	Uridine 5’-monophosphate	-
glycerol	-	thymidine	-	D-Fructose-6-Phosphate	-
α-D-Glucose-1-Phosphate	-	D-Glucose-6-Phosphate	-	D-L- α-glycerol phosphate	-

### Effect of different carbon sources for endoglucanase production

**Figure 2:**
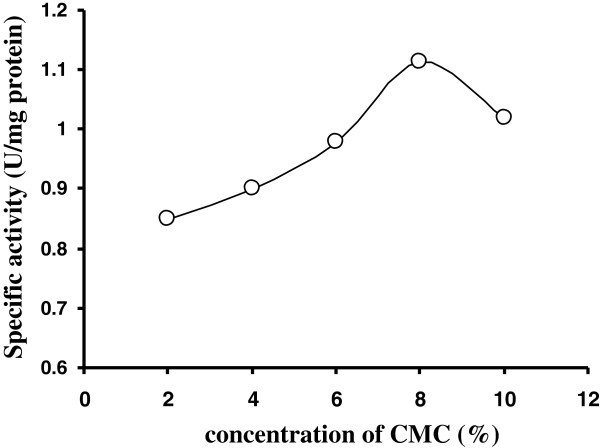
**CMCase of crude enzyme produced by culture medium containing different concentrations of CMC at 37°C after 8 d by*Bacillus*sp. Symbols: open circles, CMCase.**

Cellulase production was found to be dependent upon the nature of the carbon source used in culture media. The choice of a cheapest and appropriate substrate is of great importance for the successful production of enzymes. It was found that the strain could grow in all the substrates used but CMC promoted yield maximally when compared to agricultural waste or paper waste (Table 2). It is assumed that this is due to the less complexity and hence easy assimilation of it by the isolated microbe (Wood and Bhat 1988). Among the agricultural waste groundnut, paddy straw, banana pseudostem, sugarcane baggasse, coconut fiber were shown much low in enzymes production but orange scale showed comparatively better for enzymes production. However, newspaper waste showed significant production of enzymes. Though carboxymethyl cellulose exhibited extensive role to enzyme yield and production, the agricultural wastes (orange scale, sugarcane baggasse) may also be utilized for enzyme production as cheaper carbon source. The CMC was further tested at different concentrations for cellulase production by the isolate C1 strain and found 8% CMC was optimum for cellulase production maximally (Figure 2).

**Table 2:** **Effect of different carbon sources 1% (W/V) on cellulase production by*Bacillus*sp after 8 d of incubation at 37°C**

Agricultural waste (1% W/V)	CMCase	Avicelase	FPase	Β-glucosidase
	(U/mg protein)	(U/mg protein)	(U/mg protein)	(U/mg protein)
Paddy straw	0.111 ± 0.005	0.197 ± 0.008	0.133 ± 0.005	0.111 ± 0.005
Sugarcane baggasse	0.177 ± 0.0152	0.213 ± 0.010	0.170 ± 0.0152	0.119 ± 0.005
Banana seudo stem	0.130 ± 0.005	0.135 ± 0.0152	0.150 ± 0.0152	0.135 ± 0.0152
Groundnut scale	0.07 ± 0.008	0.07 ± 0.008	0.08 ± 0.0115	0.03 ± 0.0057
Coconut fruit fibre	0.153 ± 0.0152	0.153 ± 0.0152	0.186 ± 0.0152	0.108 ± 0.0115
Orange scale	0.54 ± 1.528	0.59 ± 1.527	0.62 ± 0.0115	0.60 ± 0.015
Whatman filter paper	0.52 ± 0.011	0.50 ± 0.011	0.51 ± 0.011	0.52 ± 0.011
Newspaper	0.65 ± 0.577	0.60 ± 0.015	0.70 ± 0.0185	0.72 ± 0.010
Printing paper	0.50 ± 0.011	0.52 ± 0.011	0.48 ± 1.154	0.45 ± 0.010
Foolscape paper	0.45 ± 0.010	0.42 ± 0.0152	0.50 ± 0.011	0.42 ± 0.0152
CMC	0.73 ± 0.02	0.77 ± 1.201	0.84 ± 0.015	0.93 ± 1.527
Avicel	0.26 ± 0.0152	0.45 ± 0.010	0.32 ± 1.527	0.26 ± 0.0152

The results of one-way factorial ANOVA reveals the amount of enzymes synthesized are in different C-sources varied significantly. F value is significant at P < 0.01 level.

### Extraction and partial purification of extracellular enzymes

Extraction of the crude enzyme was carried out by ammonium sulphate precipitation after standardization and maximum activity was recorded at 40-80% saturation. The precipitate was centrifuged, dissolved in acetate buffer (0.02 M, pH 5.2) and dialyzed. It was found that of 40-60% ammonium sulphate saturation are common for purification of bacterial cellulase (Sudan and Bajaj 2007). However, cellulase of *Pseudomonas fluorescens* was purified at 90% ammonium sulfate saturation (Bakare et al. 2005) while in *Thermomonospora*, the cellulase was precipitated by using fractional ammonium sulfate 30-88% (George et al. 2001).

### DEAE-Sepharose chromatography

Dialyzed enzyme solution from ammonium sulfate precipitation steps was subjected to ion exchange chromatography on DEAE-Sepharose column. The dialyzed proteins of crude enzyme extract were separated in different eluted fractions. All the fractions are assayed for endoglucanase activity by DNS method and it was found that Fraction (E5) shown maximum endoglucanase activity (68.1 U/mg). A brief summary of the purification steps was presented in Table 3. The specific activity of enzyme was sequentially increased at each purification step and final increment was more than 8.0 fold. Cellulase was purified 9.06 folds from *Bacillus* strain M-9 with DEAE-cellulose chromatography (Bajaj et al. 2009) and in *Cellulomonas* sp. YJ5, the enzyme was purified 17.5 fold with sephacryl S-100 chromatography (Yin et al. 2010).

**Table 3:** **Summary of purification of endoglucanase from*Bacillus*sp MTCC 10046**

Sample	Volume (ml)	Total protein	Protein conc. (mg/ml)	Total Activity (units)	Yield (%)	Specific activity (unit/mg)	Purification (fold)
Crude	70	14.2 mg	0.202	113.6	100	8.0	1
40-80% dialyzed fraction	15	1.48 mg	0.098	68.37	60.2	46.2	5.8
DEAE column E5	6	0.66 mg	0.11	44.9	39.5	68.1	8.5

*Unit* = release of reducing sugar in μmoles/ml/min.

### SDS-PAGE and molecular weight determination

**Figure 3:**
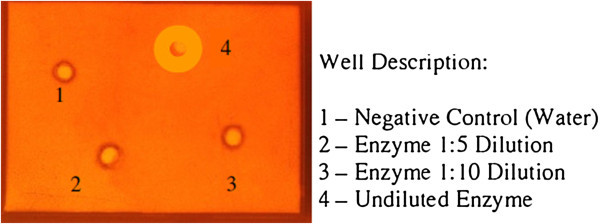
**Congo red assay on agarose slab gel for elution fraction 5 (E5) shows endoglucanase (CMCase) activity.**

**Figure 4:**
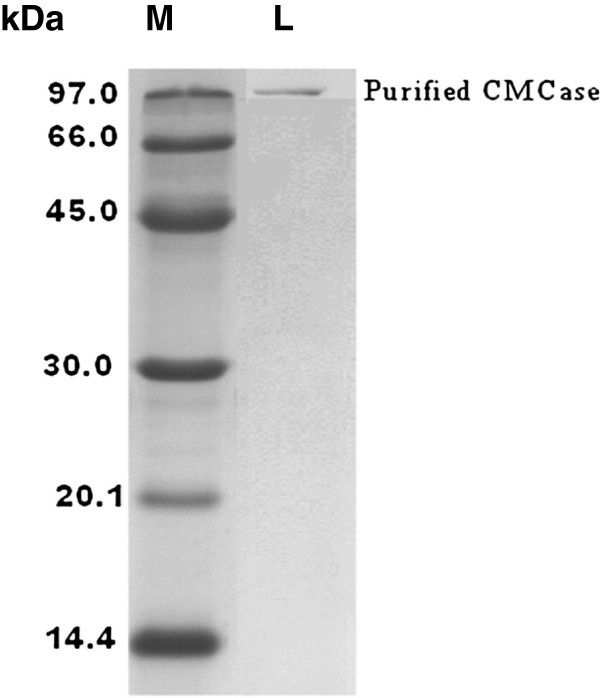
**SDS-PAGE analysis of endoglucanase purified from*Bacillus*sp MTCC 10046.** M- Indicates molecular weight markers, and L- shows purified endoglucanase band of molecular weight 97 kDa.

The endoglucanase activity was confirmed by Congo red assay through agarose slab gel method (Figure 3). Cellulase fractions from DEAE-Sepharose column with highest activity were pooled, subjected to SDS-PAGE and the molecular weight (MW) was determined and it was 97 kDa (Figure 4). The molecular weight of endoglucanase varies with different bacteria. The MW was similar with some other high MW endoglucanases like alkalophilic *Bacillus* sp. HSH-810 where it is 80 kDa (Kim et al. 2005) and *Sinorhizobium fredii* sp. strain CCRC 15769 it is 94.0 kDa (Chen et al. 2004). In *Pseudomonas fluorescens* it is 36 kDa (Bakare et al. 2005) while in *Bacillus* strains it is 54 kDa (Lee et al. 2008, Bajaj et al. 2009). But in *Thermomonospora* and *Cellulomonas* sp. YJ5 it is 38 kDa (George et al. 2001), and 43.7 kDa (Yin et al. 2010) respectively.

### Determination of K_m_ value of purified endoglucanase

**Figure 5:**
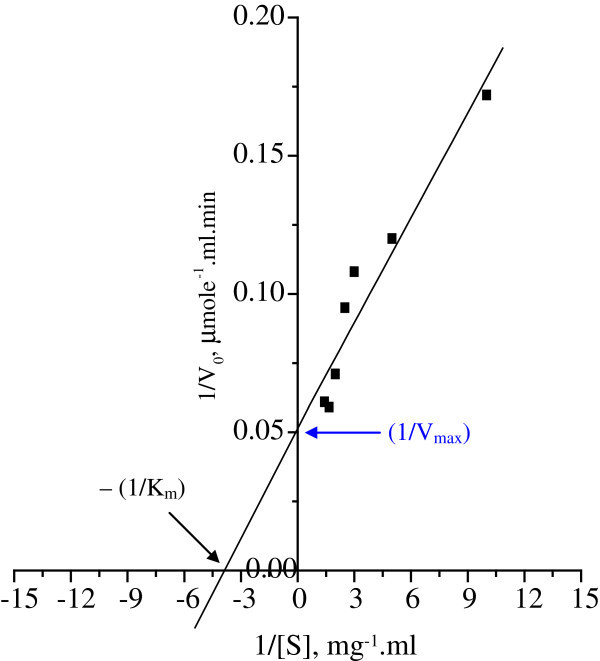
**Lineweaver- Burk double reciprocal plots for the determination of Km value of purified extracellular cellulase from*Bacillus*sp MTCC 10046.**

K_m_ value of purified extracellular cellulase was determined by Lineweaver Burk double reciprocal plot at varying substrate (CMC) concentration and found as 0.25 mg/ml. The V_max_ and k_Cat_ were found 20 μmole.ml^-1^·min^-1^ and 0.55 s^-1^ respectively (Figure 5). The activity of endoglucanase was greatly influenced by the substrate concentration in this organism. The K_m_ value of cellulase was found 3.6 mg/ml in *Pseudomonas fluorescens* (Bakare et al. 2005), as 4.97 mg/ml in *Actinobacteria anitratus* and 7.90 mg/ml in *Branhamella* sp (Ekperigin 2007). It is difficult to explain difference of K_m_ values of insoluble substrate like cellulose, however, it may be due to the isolates are of different source. The K_m_ value denotes the amount of substrate needed to achieve half the maximal initial reaction velocity (Tong et al. 1980) and is a measure of the apparent affinity of an enzyme for its substrate.

### Effect of various chemicals on endoglucanase activity

**Figure 6:**
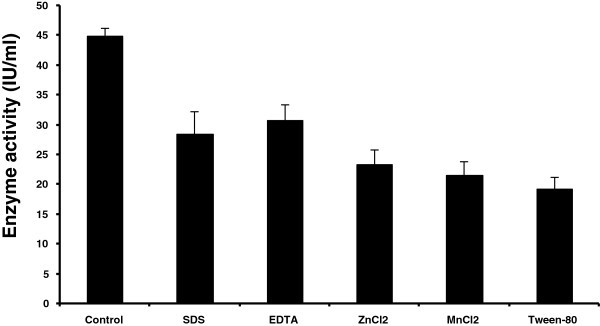
**Effect of various chemicals on enzyme (endoglucanase) activity.**

The effects of various metal ions did not show enhancement of enzyme activity of this strain (Figure 6). But, one unifying finding was that these additives inhibited enzyme activity to a certain level only. This indicated robust nature of the enzyme desirable for industrial application. Such type of enzyme inhibition was also noted in B*acillus* sp (Bajaj et al. 2009) and in *Pseudomonas fluorescens* (Bakare et al. 2005).

### Effect of temperature on endoglucanase activity

**Figure 7:**
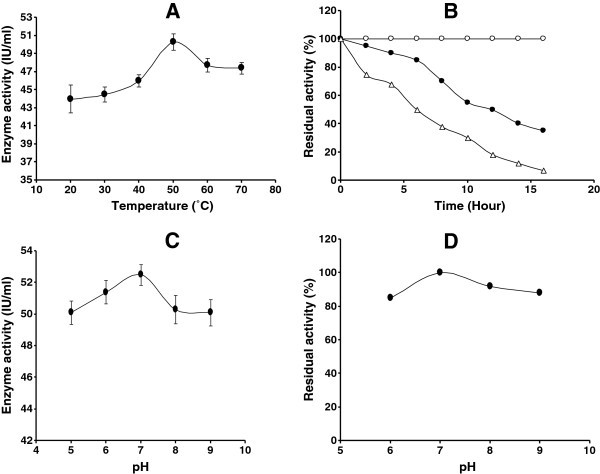
**A Effect of temperature on endoglucanase activity.** Symbol: closed circles, endoglucanase activity. **B** Thermal stability of endoglucanase: 5 IU of endoglucanase were incubated in 0.02 M acetate buffer (pH 7) at 50°C, 60°C and 70°C for different intervals and residual activity was determined. Symbol: endoglucanase activity at 50°C (open circle), endoglucanase activity 60°C (closed circle) and endoglucanase activity 70°C (open triangle). **C** Effect of pH on endoglucanase activity. Symbol: closed circles, endoglucanase activity. **D** The stability of endoglucanase in different pH at 50°C. Symbol: closed circles, endoglucanase activity.

Purified enzyme preparation was recorded to show activity over a broad range of temperature (20°-70°C) with the optimal activity at 50°C and declined thereafter (Figure 7A). Thermo-stability range of the enzyme showed that it was thoroughly stable at 50°C. However, activity of this enzyme gradually declined with increase of temperature from 60 – 70°C (Figure 7B). Nevertheless, sufficient activity of the enzyme (more than 75–80%) was present at 60 – 70°C for 1–2 h. This kind of stability of enzyme might comply with the industrial process requirements it possessed prolonged stability under high temperature. The optimum temperature of cellulase was lower than some of other *Bacillus* strains as 60^0^C in M-9 (Bajaj et al. 2009), 65°C in CH43, 70°C in RH68 (Mawadza et al. 2000), and was similar to those from other *Bacillus* strains 0-50°C (Mawadza et al. 2000), in *Bacillus amyoliquefaciens* DL3 (Lee et al. 2008) and in *Thermomonospora* (George et al. 2001). But the endoglucanase of *Bacillus licheniformis* C108 was highly stable up to 100°C (Ashabil et al. 2011) and shown better stability than that of other *Bacillus* sp (Kim et al. 2005). The cellulase of *Brevibacillus* sp strain JXL had high thermotolerance by retaining more than 50% activity at 100°C after 1 hr (Liang et al. 2009) but alkalotolerant *Nocardiopsis* sp. KNU retaining 55–70% activity at 80^0^C in pH 5.0 (Saratale and Oh 2011).

### Effect of pH on endoglucanase activity

The enzyme hydrolyzed CMC in the pH range of 5.0-9.0, and exhibited highest activity at pH 7.0 (Figure 7C). However, significantly high activity was also recorded on either side of this point which indicated that the enzyme has characteristically broad range of pH activity. The enzyme also had a broad range of pH (6.0 – 9.0) stability where it retained more than 80% activity for 1.0 hr, though maximum stability was found at pH 7.0 (Figure 7D). Similar optimum enzyme activity was also found at pH 7.0 in *Pseudomonas fluorescence* (Bakare et al. 2005), *Bacillus amyoliquefaciens* DL3 (Lee et al. 2008). But lower optimum pH (5.0) for enzyme activity with broad ranges of pH stability was found in *Thermomonospora* (George et al. 2001), and in *Bacillus* strain M-9 (Bajaj et al. 2009). Cellulase of *Bacillus licheniformis* was found to be more stable under acidic condition (Bischoff et al. 2006), however, it was optimally active at alkaline pH in *Bacillus* sp. HSH-910 (Kim et al. 2005) and in *Bacillus sphaericus* JS1 (Singh et al. 2004).

### Stability of endoglucanase in the presence of commercial detergents

**Figure 8:**
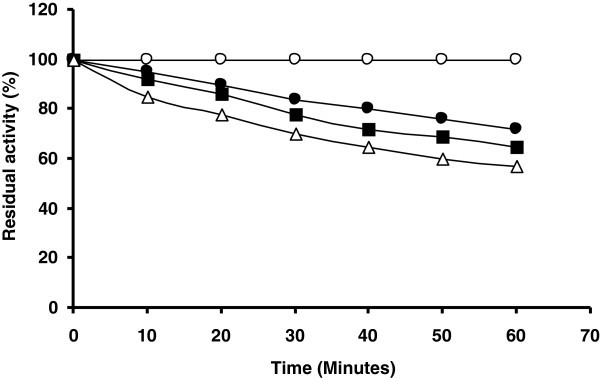
**Stability of cellulase in various commercial detergents: endoglucanase was incubated at 50°C in the presence of various detergents and samples were removed every 10 min for 1 h and endoglucanase activity was determined: endoglucanase activity in control (without detergents) (open circle), endoglucanase activity in Ariel (closed circle), endoglucanase activity in Surf Excel (closed square) and endoglucanase activity in Tide (open triangle).**

The endoglucanase of this *Bacillus* sp was also stable in different commercial detergents. The enzyme showed maximum stability in the presence of Ariel, having residual activity of 72% after incubation at 50°C for 1 hr (Figure 8). The enzyme had 65% and 57% residual activities in the presence of Surf Excel and Tide respectively under similar condition (Figure 8). Data are in accordance with the results of *Thermonospora* sp (George et al. 2001). The alkalophilic *Bacillus* strain producing alkaline cellulase with pH optima in the range of 8.5 – 9.5 having industrial application as laundry detergent additives (Shikata et al. 1990). Although this *Bacillus* sp. was capable of growing in neutral pH but it remain stable in broad range of pH 6.0 -9.0 and in some detergents. Among the useful properties of the *Bacillus* cellulases *per se* are that many are active and stable over a wide range of pH and temperature (Lee et al. 2008).

## Conclusions

It may be concluded that the organism *Bacillus* sp. MTCC 10046 has the potential to produce thermostable endoglucanase with broad range of pH and temperature stability which could have potential applications for wide range of industries. Industrial processes are generally carried out at elevated temperatures; therefore enzymes with high temperature optima and their stability are desired for industrial applications (Viikari et al. 2007). However, further investigation is needed in depth on optimization of cost effective substrate for bulk production of enzymes and molecular basis of thermostability.

## Methods

### Isolation and screening of cellulolytic bacteria

The bacterial strains were isolated from cow dung by serial dilution on Omeliansky’s agar medium (Omeliansky 1902) [g/L (W/V), (NH_4_)_2_SO_4_ 1.0; K_2_HPO_4_ 1.0; MgSO_4_.7 H_2_O 0.5; NaCl traces; carboxymethyl cellulose (CMC) 1%, pH 7.0]. Preliminary screening of cellulase producing isolates was carried out on CMC agar plates as mentioned by Teather and Wood (Teather and Wood 1982). Twenty bacterial isolates were selected out of which one (designated C1) with highest CMCase activity was finally selected. The strain C1 was identified as *Bacillus* sp. by full 16 S rDNA sequence homology and deposited to Microbial Type Culture Collection Centre and Gene Bank with accession number MTCC 10046.

### DNA extraction and molecular phylogenetic analyses using 16S rRNA gene sequence

Genomic DNA was extracted according to Marmur (Johnson 1994). PCR amplification of the 16S rRNA gene was carried out using universal primers 8–27f and 1492r and amplified PCR product was purified and sequenced as described by Saha and Chakrabarti (2006). A continuous stretch of 1514-nucleotide long gene sequences of 16S rRNA gene was used to search for similar sequences from RDP database Release 10 http://rdp.cme.msu.edu/) using various online tools (CLASSIFIER, SEQMATCH). After confirmation of generic affiliation, sequences from type strains of different species were retrieved from GenBank. All these sequences were aligned by CLUSTAL_X programme (Thompson et al. 1997) and edited manually. Similarity values were determined after pair wise alignment by CLUSTAL_X programme followed by manual calculation. A phylogenetic tree showing relationship between C1 and other reference strains was constructed by neighbor-joining (NJ) method with Jukes and Cantor correction using TREECON software as described by Saha and Chakrabarti (2006).

### BIOLOG Method

Suspension of active bacterial culture (Grown on TSA at 25 C° for 72 hours) of defined density (as per manufacture’s instruction) was inoculated into microtitre plate. An inoculum of 150 μl was dispensed into Biolog GN2 Microplates using a multichannel micropipette. The oxidation of various carbon sources as indicated by reduction of tetrazolium violet dye that results in production of purple color was monitored and recorded by using a micro plate reader that is coupled to a computer. The later has software for comparison of various patterns of oxidation as per Biolog database.

### Preparation of agricultural and paper waste used as carbon source for CMCase production

The Banana agro waste (pseudo stem, leaves, etc.) used for saccharification were freshly collected. The waste was washed thoroughly with water and air dried. It was ground to powder using electric grinder and sieved. Orange fruit was washed thoroughly with water, peeled and sliced. The juice was removed with the aid of a squeezer and the pulp separated from the pericarp (albedo) and the three materials were sun-dried separately. They were later oven-dried at 70°C, still being handle separately and then pounded using a mortar and pestle. All waste substrates then ground using a blender. Raw rice straw (RS) was obtained from local farmers. It was cut to 1–2 cm length and washed thoroughly with tap water until the washings were clean and colorless and then dried in a oven at 65°C to constant weight. Oven dried RS was then ground with electric grinder and was used in the experiments. Sugarcane baggasse also prepared in the same method. Papers were cut to 1 × 6 cm length and were used in the experiment.

### Optimum concentration of CMC

We find that among different carbon sources CMC was the best carbon source. So, to find out the suitable concentration of CMC, different concentrations of CMC was tested.

### Enzyme separation

The isolate C1 was inoculated in 100 ml of Omeliansky medium (with 8% CMC as carbon source) and was grown at 50°C for 7 days. Cell separation was made by centrifugation of fermented broth at 10,000 × g for 20 min and the cell free broth used as crude enzyme.

### Partial purification of endoglucanase

Crude enzyme preparation was obtained after cultivation of the bacteria under submerged fermentation in shake flask condition. After centrifugation of the fermented broth the supernatant was subjected to ammonium sulphate precipitation with continuous stirring at 4°C. The supernatant was precipitated with ammonium sulphate at 40-80% saturation for two hours with gentle stirring. The precipitated proteins were recovered by centrifugation at 8000 rpm for 20 min and were dialyzed against 0.02 M sodium acetate buffer (pH 5.2) for 24 h. The dialyzed solution was then applied to DEAE-Sepharose column.

### DEAE-Sepharose chromatography

The semi-purified enzyme solution was subjected to DEAE-Sepharose column. The 35 ml of the enzyme solution was applied to DEAE-Sepharose (Sigma-aldrich.) column which was previously equilibrated with 0.02 M Acetate buffer (pH 5.2). The entire purification was carried out at 4°C. The column was first washed with equilibration buffer, and then bound proteins were eluted using linear gradient of 0.05 -1.0 M NaCl (in acetate buffer) at a flow rate of 1 ml/minute. The elute fractions (3.0 ml) was by an automatic fraction collector and monitored for enzyme activity as well as for protein concentration at 280 nm. After step-wise eluted each fraction from DEAE-Sepharose column, the total activity and specific activity of endoglucanase was assayed by DNS method, the fraction showing high activity were pooled and used for SDS-PAGE analysis.

### SDS-PAGE and determination of molecular weight of endoglucanase

The active eluted fraction (E5 = 5^th^ eluted fraction) collected from DEAE-Sepharose chromatography was used for analysis by SDS-PAGE (Robyt and White 1990). Resolving gel consisted of 12% polyacrylamide in Tris–HCl (1.5 M, pH 8.8), while stacking gel consisted of 4.5% polyacrylamide in Tris–HCl (0.5 M, pH 6.8). Agarose slab gel method was used for congo red assay. An agarose slab gel (0.5% W/V) was prepared containing 0.2% (W/V) CMC as substrate for the enzyme assay. Four slots were punched using a gel puncher and the different dilutions (1:5, 1:10 and undiluted) of fractions E5 and negative control were loaded in it. The slab was placed overnight in a moist chamber at room temperature for diffusion, followed by flooding with 0.5% aqueous congo red solution and de-staining of slab with 2 M NaCl solution and recorded as photo image for further confirmation.

### Determination of protein concentration

Concentration of protein was determined following the method of Lowry et al. (1951) using BSA as standard and the protein in column elute fraction was also monitored by spectrophotometrically at 280 nm.

### Assay of endoglucanase activity

The activity of cellulase was assayed by incubating 1 ml of reaction mixture consisting of 0.5 ml of 1% CMC in 0.02 M Sodium acetate buffer, pH 5.2 and 0.5 ml of suitably diluted enzyme solution incubated at 40°C for 1 hr. Enzyme and reagent blanks were incubated maintaining the same condition simultaneously. The amount of reducing sugar released was determined by dinitrosalicylic acid (DNS) method (Miller 1959). One International Unit (IU) of enzyme activity for endoglucanase was defined as the amount of enzyme releasing 1 μmol of reducing sugar from CMC per minute. The specific activity determined as the number of units of enzyme activity per milligram of enzyme protein.

### Determination of Km value

The Michaelis-Menten constant (Km) of purified extracellular cellulase of strain C1 was determined by varying the concentration of CMC in 0.02 M acetate buffer, pH 5.2. The kinetic parameters were determined from Lineweaver Burk double reciprocal plot (Lineweaver and Burk 1934). The initial velocity measured by quantitatively measuring the amount of one of the product at various time intervals (Robyt and White 1990).

### Effect of various chemicals on endoglucanase activity

Enzyme activity measured in the presence of detergents (SDS, Tween-80), metal ions (MnCl_2_, ZnCl_2_) and EDTA at a concentration of 10 mM.

### Effect of temperature on endoglucanase activity

Enzyme activity was measured by treating the enzyme mixture at various temperatures, ranging from 20 to 70°C.

### Effect of pH on endoglucanase activity

The activity was measured at various pH (5.0-9.0), maintained using different buffering system.

### Effect of pH and temperature on stability of the enzyme

The effect of pH on stability of the enzyme was determined by incubating 5 IU of enzyme for 1 h. at 50°C, in a buffer of desired pH before addition to the reaction mixture; similarly the effect of temperature was determined by treating the enzyme at varying temperatures before addition to the reaction mixture.

### Stability of endoglucanase in commercial detergents

The stability of endoglucanase in the presence of the commercial detergents such as Ariel, Surf Excel and Tide was investigated by incubating the enzyme in the presence of the detergent (7 mg/ml) at 50°C. Aliquots of enzymes were removed at intervals of 10 min. and the residual activity of the enzyme was determined using standard assay conditions.

### Statistical analysis

All the data generated in the study are the mean ± SEM of 3 replicates. All data were subjected to student’s t- test analysis with significance level of P < 0.01 using SPSS software package.
